# Improvement of Ethanol Tolerance by Inactive Protoplast Fusion in *Saccharomyces cerevisiae*

**DOI:** 10.1155/2020/1979318

**Published:** 2020-01-20

**Authors:** Yi Xin, Mei Yang, Hua Yin, Jianming Yang

**Affiliations:** ^1^State Key Laboratory of Biological Fermentation Engineering of Beer, Technology Center of Tsingtao Brewery Co., Ltd., Qingdao, Shandong 266061, China; ^2^Single-Cell Center, CAS Key Laboratory of Biofuels and Shandong Laboratory of Energy Genetics, Qingdao Institute of BioEnergy and Bioprocess Technology, Chinese Academy of Sciences, Qingdao, Shandong 266061, China; ^3^Energy-rich Compounds Production by Photosynthetic Carbon Fixation Research Center, College of Life Sciences, Qingdao Agricultural University, Qingdao, Shandong 266109, China

## Abstract

*Saccharomyces cerevisiae* is a typical fermentation yeast in beer production. Improving ethanol tolerance of *S. cerevisiae* will increase fermentation efficiency, thereby reducing capital costs. Here, we found that *S. cerevisiae* strain L exhibited a higher ethanol tolerance (14%, v/v) than the fermentative strain Q (10%, v/v). In order to enhance the strain Q ethanol tolerance but preserve its fermentation property, protoplast fusion was performed with haploids from strain Q and L. The fusant Q/L-f2 with 14% ethanol tolerance was obtained. Meanwhile, the fermentation properties (flocculability, SO_2_ production, *α*-N assimilation rate, GSH production, etc.) of Q/L-f2 were similar to those of strain Q. Therefore, our works established a series of high ethanol-tolerant strains in beer production. Moreover, this demonstration of inactivated protoplast fusion in industrial *S. cerevisiae* strain opens many doors for yeast-based biotechnological applications.

## 1. Introduction

Beer has been consumed for long time all over the world. It is produced from barley by fermentation with yeast *Saccharomyces cerevisiae*, which is the best ethanol producer among many fermentative microorganisms. Although yeast cells have developed appropriate mechanisms to deal with several types of damages caused by increased ethanol concentration, excessive ethanol causes inhibition of its own production, leading to growth arrest and eventually cell death [1–3]. Therefore, strong improvement of yeast tolerance to high levels of ethanol would be beneficial for the cost-competitive bioethanol production. However, many approaches such as ferment optimization [4], adaptive evolution [5, 6], and genetic engineering [7, 8] could not effectively improve ethanol tolerance in yeast [9]. Meanwhile, intrinsic fermentation properties were usually degenerated in industrial strains after abovementioned approaches [10]. Moreover, genetic engineering could result in unforeseeable transgenic risk for human health. Therefore, a safe, stable, and efficient technology is a requisite for large enhancement of yeast ethanol tolerance.

One approach to achieve improved properties of ethanol tolerance is protoplast fusion, which involves the generation of recombinant by allowing recombination between genomes of different parental strains; this is followed by selection to combine the beneficial alleles of the parental isolates into single cells showing the favorite therapeutic enzymes with desired characters [11–13]. In the production of Chinese rice wine, strategies of protoplast fusion were conducted to obtain yeast diploid hybrids with excellent oenological characteristic. The flavor profiles in Chinese rice wine were improved by using a yeast diploid hybrid, which exhibited higher ethanol tolerance and fermentation activity than the original diploid parents [6]. However, the method need some more optimizations to improve the screening efficiency. To solve this problem, parental inactivated protoplast fusion is considered to be a robust approach to readily obtain fusants. Compared with other screening methods such as nutrient deficiency type and drug resistance, parental inactivated protoplast fusion has significantly improved the screening efficiency for fusion recombinants. So far, the inactivated protoplast fusion had been applied in many organisms, such as *Solanum tuberosum* [14], *Candida albicans* [15], *Bacillus subtilis* [16], *Saccharomyces cerevisiae* [17], and *Monascus purpureus* [18]. In this way, diploids were formed and the diploids were verified to be stably genetic in some fungi [15, 17, 18]. Meanwhile, unfused protoplasts and homokaryotic fusion products will not regenerate. Hence, creating yeast fusants by parental inactivated protoplast fusion, which the genomes of different strains were contained within one cell, to obtain yeast with superior ethanol tolerance was very significant in the beer industry.

Acquiring yeast with high ethanol tolerance was always desirable to brewers, which could theoretically result in more complete fermentation and higher production quality of beer. Fermentative strain Q is frequently used as the beer producer. In the present study, we found that *S. cerevisiae* strain L exhibited a higher ethanol tolerance (14%, v/v) than the fermentative strain Q (10%, v/v). In order to enhance the strain Q ethanol tolerance but preserve its fermentation property, protoplast fusion was performed with haploids from strains Q and L. Meanwhile, to optimize the current methods on parental inactivated protoplast fusion, many procedures such as haploid preparation, protoplast formation, and regeneration were optimized by setting gradients on the incubated medium, duration, temperature, enzyme concentration, etc. The fusant Q/L-f2 with 14% ethanol tolerance was finally obtained. Moreover, the fermentation properties (flocculability, SO_2_ production, *α*-N assimilation rate, GSH production, etc.) of Q/L-f2 were similar to those of strain Q. Therefore, our works established one high ethanol-tolerant strain in beer production. Moreover, this demonstration of inactivated protoplast fusion in industrial *S. cerevisiae* strain opens many doors for yeast-based biotechnological applications.

## 2. Materials

### 2.1. Yeast Strains, Medium, and Reagents

**Figure 1:**
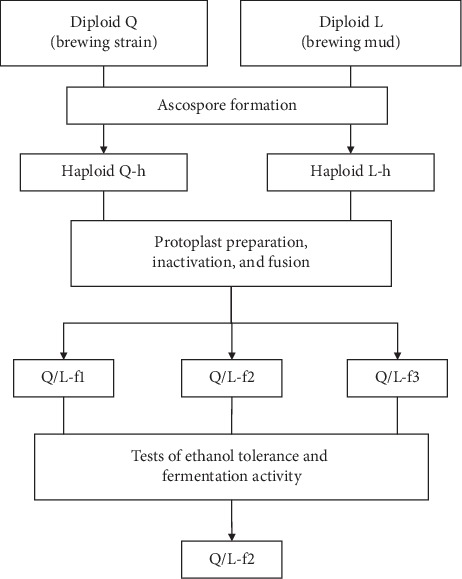
Systematic diagram of producing and screening procedure for Q/L fusants.

As shown in Figure 1, diploid *S. cerevisiae* strains Q (the brewing strain) and L (the isolated strain from brewing yeast mud) were kindly gifted by the beer corporation. Since tetraploid strains (which might be produced by the fusion of diploids) are not genetically stable because of the high chromosome number, haploid strains were necessary for the following fusion [19]. Thus, haploid strains of Q-h and L-h were generated by ascospore formation for protoplast fusion. Then fusants Q/L-f1, Q/L-f2, and Q/L-f3 were selected as candidates for the tests of ethanol tolerance and fermentation activity.

YPD medium (2% glucose, 2% peptone, and 1% yeast extract) was used for yeast growth. YPK medium (1% potassium acetate, 2% peptone, and 1% yeast extract), HS medium (1% potassium acetate, 0.1% yeast extract, 0.05% glucose, 0.005% adenosine, 0.005% uracil, 0.01% tryptophan, 0.01% leucine, and 0.01% histidine), McClary medium [20], Kleyn medium [21], and SPM medium (0.025% yeast extract, 0.01% glucose, 0.1% KCl, and 5 mmol/L KH_2_PO_4_) were used for yeast sporulation. For preparing protoplasts, RM medium (YPD containing 0.6 mol/L KCl and 0.025 mol/L CaCl_2_) was used as regeneration medium. Malt extract broth used for fermentation was adjusted to 10–12% brix. All reagents and standards were purchased from Sigma Aldrich.

### 2.2. Preparation of the Yeast Haploids and Protoplasts

For haploid preparation, 5 ml diploid *S. cerevisiae* strains Q-h and L-h (1 × 10^8^ cfu/mL) were centrifuged with 3500 r/min for 10 min at room temperature and washed three times in sterile cold water. The suspensions were inoculated into 20 mL YPK medium for 24 h at 28°C in a rotary shaker (200 r/min). The cells were collected and washed three times in normal saline. The suspensions were inoculated into 20 mL HS medium, McClary medium, Kleyn medium, or SPM medium. The spore formation was then surveyed under the microscope and the formation rate was calculated as [A/(A + B)] × 100% (A: spore number; B: diploid number). As for the ascospore release, spore-containing *S. cerevisiae* solution was centrifuged at 3000 r/min, 10 min for cell collection. The sediment was resuspended with 5 mL softening buffer (10 mM dithiothreitol, 100 mM Tris/H_2_SO_4_, pH 9.4) containing 0.1–0.3 g/L lyticase (from *Arthrobacter luteus*, ≥2000 U/mg, sigma Aldrich, USA) and incubated in 30°C for 30 min. Then, the ascospores were stained by Meran carbonate (Solarbio) (haploid: pink; diploid: blue) [22]. The spores were incubated in 60°C for 10 min and collected via centrifugation. After suspension, the spores were inoculated into 20 mL YPD medium for 24 h at 30°C in a rotary shaker (100 r/min). Then, the haploids Q and L were screened in malt extract broth with 12% ethanol.

For protoplast preparation, the method described by Curran and Bugeja was applied [23]. Haploid strains Q and L were preactivated (i.e., the strains were previously activated for the following inoculation) and inoculated into 50 mL YPD medium for 16 h at 30°C in a rotary shaker (180 r/min). Suspensions (1 × 10^8^ cfu/mL) of the two stains were centrifuged with 3000 × g for 5 min at room temperature and washed twice in sterile cold water. Cells were resuspended in 4 mL of PB buffer (0.01 M Tris-HCl, pH 6.8, 20 mM MgCl_2_, 0.5 M sucrose) with 0.01 M *β*-mercaptoethanol and incubated for 30 min in a rotary shaker (150 r/min) at 30°C. Cells were then washed, resuspended in 10 mL of PB buffer containing with 2% snailase (Solarbio), and incubated in water bath at 30°C. Protoplasts were washed and suspended in PB buffer for further use.

### 2.3. Protoplast Inactivation and Fusion

The inactivation of protoplasts results in the loss of the original activity of many enzyme proteins in the cytoplasm, whereafter inducing the disability of independent protoplast regeneration [24]. For protoplast inactivation, 5 ml Q and L protoplast suspensions were incubated at 60°C for 5, 10, 15, 20, 25, or 30 min. Meanwhile, another 5 ml Q and L protoplast suspensions were irradiated by UV for 2, 4, 6, 8, 10, 12, or 14 min. Then, the treated fluid was spread onto RM plates. The clones were then counted under the microscope, and the survival rate was calculated as A/B × 100% (A: clone number under inactivation; B: clone number without inactivation).

For protoplast fusion, equivalent amounts (1 × 10^8^ cfu/mL) of heat- or UV-inactivated protoplast Q and L were mixed, centrifuged, and treated with 2 mL of 35% polyethylene-glycol (molecular weight 6000) and 100 mM CaCl_2_ for 30 min in a rotary shaker (100 r/min) at 30°C. Cells were centrifuged (3000 × g during 5 min at room temperature) and washed twice with PB buffer. Appropriate dilutions of cells in PB buffer were spread onto RM medium. After incubating at 30°C for 4 days, the observed colonies were purified on the same medium.

### 2.4. Ethanol Tolerance Assay and Determination of Fermentation Properties

To evaluate ethanol tolerance of yeast strains, 100 *μ*l precultivated yeast cells (1 × 10^8^ cfu/mL) were inoculated into 10 ml malt extract broth plus Durham's fermentation tube. The ethanol concentrations of malt extract broth are 0%, 6%, 8%, 10%, 12%, 14%, and 16% (v/v). These cultures were incubated at 30°C for three days, and then the aerogenesis and growth rate were measured.

Fermentation activity could be determined by CO_2_ production [25], cell concentration [26], superoxide dismutase/acid phosphatase activity [27, 28], proline/malonaldehyde concentration [29, 30], relative electrolytic leakage [31], etc. Here, we used the CO_2_ production method described in previous papers with a few modifications [32–35]. As for the measurement of CO_2_ weight loss, a precultured cell suspension (1 × 10^8^ cfu/mL) was inoculated into a 250 mL Erlenmeyer flask containing 45 ml malt extract broth at ration of 10% and incubated at 28°C. Fermentative activity was monitored via measuring reduction weight of malt medium, which represents CO_2_ production. Reduction weight of malt extract broth was determined each day.

As for the measurement of SO_2_ concentration, fermentation broth was centrifuged and 2.5 ml supernatant was mixed with a 0.5 ml Mercury stabilizer, 1.25 ml H_2_SO_4_ (0.1 M), and 3.75 ml NaOH (0.1 M) and stirred for 15 second. Then, 2.5 ml H_2_SO_4_ and 14.5 ml H_2_O were added to make the total volume 25 ml. Then, 0.5 ml methanol, 0.5 ml pararosaniline hydrochloride colorant, and 1.5 ml H_2_O were added into 2.5 ml mixture. SO_2_ concentration was determined by measuring the absorbance of the suspension at 550 nm. The *α*-nitrogen was measured by the colorimetric ninhydrin method [33]. The glutathione (GSH) concentration was measured by the DTNB (5,5′-dithiobis-2-nitrobenzoic acid) method [34]. The sedimentation ability was evaluated under standard conditions, using a technique previously described [35].

## 3. Results and Discussions

### 3.1. *S. cerevisiae* Strain L Exhibited a Higher Ethanol Tolerance than Strain Q

**Figure 2:**
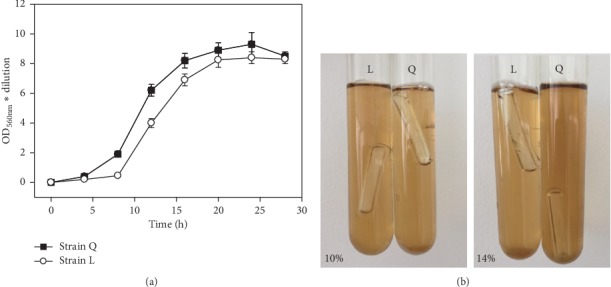
Properties of *S. cerevisiae* strains Q and L. (a) Growth kinetics of strains Q and L from 0 h to 32 h; OD_560_ value was used to indicate the growth. (b) Growth status of strains Q and L under 10% (v/v) after 3-day cultivation and 14% (v/v) ethanol concentration after 5-day cultivation.

To compare the properties of strain Q and L, the growth rate of the two strains was measured (Figure 2(a)). For strain Q and L, the log phase is from 8 h to 20 h, and the highest cell density is at 24 h, which is thus used as the moment for sampling. Meanwhile, Q and L 26S rDNA genes were amplified and sequenced. The data showed that the 26S rDNA size of both strains is ∼600 bps. Moreover, the 26S rDNA of strain Q is almost identical to the counterpart of strain L (Figure S1). Therefore, the results suggested that strains Q and L are similar in growth and genesis.

To compare the ethanol tolerance between Q and L, Durham's fermentation was used in malt extract broth containing 0%–16% ethanol (v/v). The ethanol tolerance was obtained by the rise of Durham's tube, due to gas production during the growth of yeast. The results showed that strain L exhibits a higher ethanol tolerance (14%) than the strain Q (10%) (Figure 2(b)). Therefore, the 14% ethanol concentration (v/v) was used for further screening of Q/L fusants.

### 3.2. Optimization of Haploid Preparation in Strains Q and L

**Figure 3:**
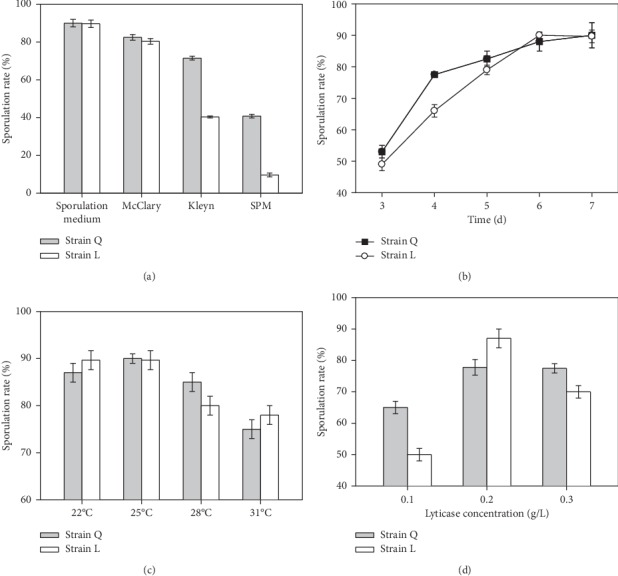
Comparison and optimization of the parameters of haploid preparation in strains Q and L. (a) Comparison of the sporulation rate among four kinds of culture mediums. ((b)–(d)) Comparison of the sporulation rate among different culture duration (b), culture temperature (c), or lyticase concentration (d). Values are presented as mean ± SD (*n* = 3).

To optimize the conditions of culture for haploid preparation, preactivated strains Q and L were inoculated into HS medium, McClary medium, Kleyn medium, or SPM medium, respectively. Then, the culture solutions were incubated in 25°C for 7 days. The HS medium exhibited the highest spore formation rate (90% for strain Q and 89.7% for strain L) among four kinds of mediums (Figure 3(a)). Moreover, strains Q and L were incubated in HS medium at 25°C for 3, 4, 5, 6, or 7 days. The spore formation rate was identified to be in proportion to the length of incubation time (Figure 3(b)). The highest spore formation rate was obtained at the 7^th^ day, probably due to the nutrient depletion. Furthermore, strains Q and L were incubated in HS medium for 7 days at 22°C, 25°C, 28°C, or 31°C. The highest spore formation rate was obtained at 25°C (Figure 3(c)). Therefore, the haploid preparation of strains Q and L was conducted in HS medium at 25°C for 7 days.

Lyticase concentration is considered to be an important parameter for haploid preparation, thus 0.1 g/L, 0.2 g/L, or 0.3 g/L lyticase was used for cell wall breaking. The effect was identified by carbol fuchsin and methylene blue dyes (haploid: pink; diploid: blue, Figures S2A and S2B). Microscopy results exhibited that the highest spore formation rate is produced by 0.2 g/L lyticase (77.8% for strain Q and 87% for strain L) (Figure 3(d)). Moreover, lyticase-treated ascospores were found to be agglutinative. Meanwhile, the ascospore release rate was found to be in proportion to the number of spores in ascus, which might be because the cell wall of ascus is much thinner than that of the single ascospore (Figures S3A and S3B). Therefore, strains Q and L were incubated by 0.2 g/L lyticase in HS medium at 25°C for 7 days to finally form the haploids Q-h and L-h.

### 3.3. Optimization of Protoplast Formation

**Figure 4:**
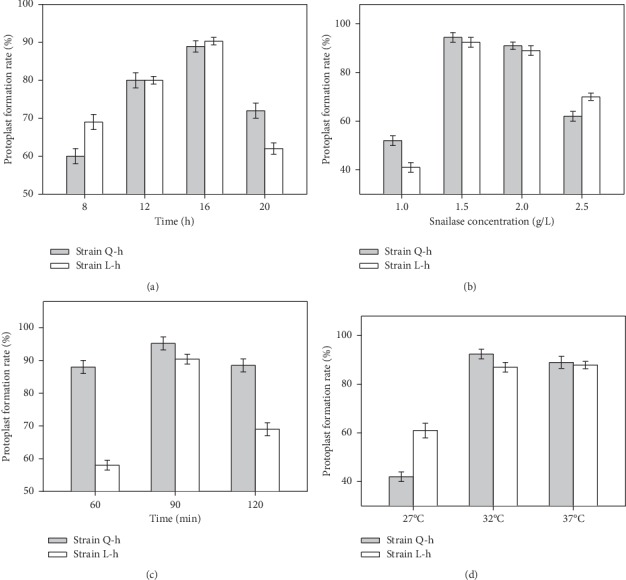
Optimization of the protoplast formation in haploids Q–h and L–h. ((a)–(d)) Comparison of the protoplast formation rate among different fungus ages (a), snailase concentration (b), incubated duration, (c) and temperature (d). Values are presented as mean ± SD (*n* = 3).

To optimize the parameters of protoplast formation, 8 h-, 12 h-, 16 h and 20 h-cultured haploid Q-h and L-h were treated by 1.5 ml 2% snailase and then incubated at 32°C for 90 min. The highest rates of protoplast preparation (88.9% for haploid Q-h and 90.3% for haploid L-h, Figure 4(a)) were obtained at 16 h-cultured samples. To explore the effect of enzyme concentration, 1 ml, 1.5 ml, 2 ml, or 2.5 ml 2% snailase was added into 16 h-cultured Q-h or L-h and then incubated at 32°C for 90 min. The results showed that solutions within 1.5 ml 2% snailase exhibited a globally better effect than other samples, with the protoplast preparation rate of 94.4% for haploid Q-h and 92.4% for haploid L-h (Figure 4(b)).

To measure the snailase digestion time, haploids Q-h and L-h were cultured for 16 h, and then 1.5 ml 2% snailase was added. The solutions were incubated at 32°C for 60 min, 90 min, or 120 min. The highest rates of protoplast preparation (95.22% for haploid Q-h and 90.4% for haploid L-h, Figure 4(c)) were obtained at 60 min-incubated samples. Moreover, 16 h-cultured haploids were treated with 1.5 ml 2% snailase and then incubated 90 min at 27°C, 32°C, or 37°C, respectively. The data exhibited that the 32°C-incubated samples could represent a globally better protoplast preparation (92.4% for haploid Q-h and 87.9% for haploid L-h, Figure 4(d)) than other samples. Therefore, the optimum condition for protoplast formation and regeneration is the addition of 1.5 ml 2% snailase into 16 h-cultured haploids, then 90 min incubation at 32°C, to produce protoplasts Q-hp and L-hp.

### 3.4. Production of High Ethanol-Tolerant Fusants

**Figure 5:**
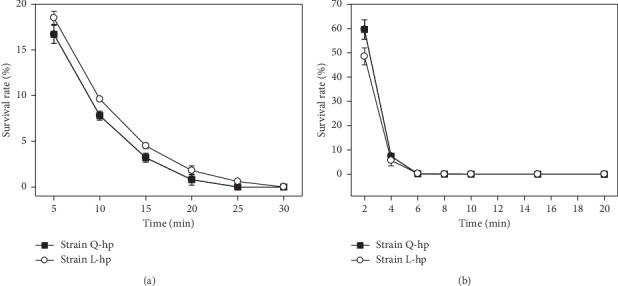
Optimization of the protoplast inactivation in protoplast Q-hp and L-hp. ((a)-(b)) Comparison of the survival rate among different incubated duration under heating (a) or UV (b). Values are presented as mean ± SD (*n* = 3).

In order to produce fusants, the protoplasts need to be inactivated in this study. Inactivation means protoplasts lose their independent regenerative activity, rather than really dead. Therefore, the inactivated protoplast is believed to be regenerated by fusion, even though its growth was not detected (i.e., full protoplast inactivation). The hyperthermal inactivation of protoplasts results in the damage of ribosome RNA, which then gives rise to the loss of the original activity of many enzyme proteins in the cytoplasm, whereafter inducing the disability of independent protoplast regeneration [24]. Successful hyperthermal inactivation depends on the temperature and duration of heating. Generally, yeast protoplast could be completely inactivated by 60°C heating for 10 min. However, enzyme-treated yeast should be activated by more heating, due to the protective effect of the high osmotic buffer. The results exhibited that the protoplast survival rate is inversely proportional to the heating duration (Figure 5(a)). To determine the inactivated condition, heating duration was appointed as the point of 0% protoplast survival rate, that is, 25 min for Q-hp and 30 min for L-hp, under 60°C.

Ultraviolet (UV) inactivation was conducted according to the previous methods [36], i.e., UV irradiation in 30 W and 30 cm separation. To explore the irradiation duration, Q-hp and L-hp were UV-inactivated for 2 min, 4 min, 6 min, 8 min, 10 min, 12 min, or 14 min, respectively. Moreover, to avoid the photoreactivation, UV-irradiated samples should be immediately put in the dark for 30 min. As shown in Figure 5(b), both Q-hp and L-hp were completely inactivated at 14 min. Therefore, the protoplasts were assigned to be inactivated under UV irradiation in 30 W and 30 cm separation for 14 min.

To produce strain Q and L fusants, inactivated protoplasts Q-hp and L-hp were fused in 40% polyethylene glycol of 6000 molecular weight (PEG6000). The mixture was regenerated for three days. Then, the colonies were screened under 14% ethanol (v/v). Three fusants Q/L-f1, Q/L-f2, and Q/L-f3 were selected as candidates for the further tests of fermentation activity.

Before this study, creating fermenting yeast with superior ethanol tolerance and fermentation activity had been conducted in other systems. For example, to improve the flavor profiles of Chinese rice wine, ethanol domestication, UV mutagenesis, and protoplast fusion were conducted to create yeast hybrids with excellent oenological characteristics. The obtained diploid hybrid F23 showed a cell viability of 6.2% under 25% ethanol. Meanwhile, the total content of flavor compounds in F23 wine was 20–26.6% higher than that of parent wines [6]. However, such technologies had not been applied in the beer industry. Moreover, the methodology system needs some more optimizations to improve the screening efficiency. In this study, heat- or UV-mediated parents inactivation was used for efficient screening of fusants in beer. The significantly improved ethanol tolerance while maintaining oenological properties of fusants demonstrated that application of inactivated protoplasts fusion method was an efficient approach to get superior industrial yeast strains.

### 3.5. High Ethanol-Tolerant Fusants Exhibited Similar Fermentation Properties with Strain Q

To evaluate the fermentation properties of Q/L fusants, we measured a series of fermentation-related parameters. As mentioned in the previous studies, CO_2_ weight loss is usually used for representation of the glycolysis rate [37]. SO_2_ could increase the stability of made-up beer [25]. Amino acid profiles (usually represent with *α*-N assimilability) could affect beer flavor via the interaction among proteins, amino acids, and polyphenol [38]. Glutathione (GSH) is considered to be useful for detoxification and delaying senescence [39]. Meanwhile, moderate sedimentation ability should be proper for beer fermentation [40]. Therefore, CO_2_ weight loss, SO_2_ content, *α*-N assimilability, GSH content, and sedimentation were used for the comparison of fermentation properties between Q/L fusants and their parents.

**Figure 6:**
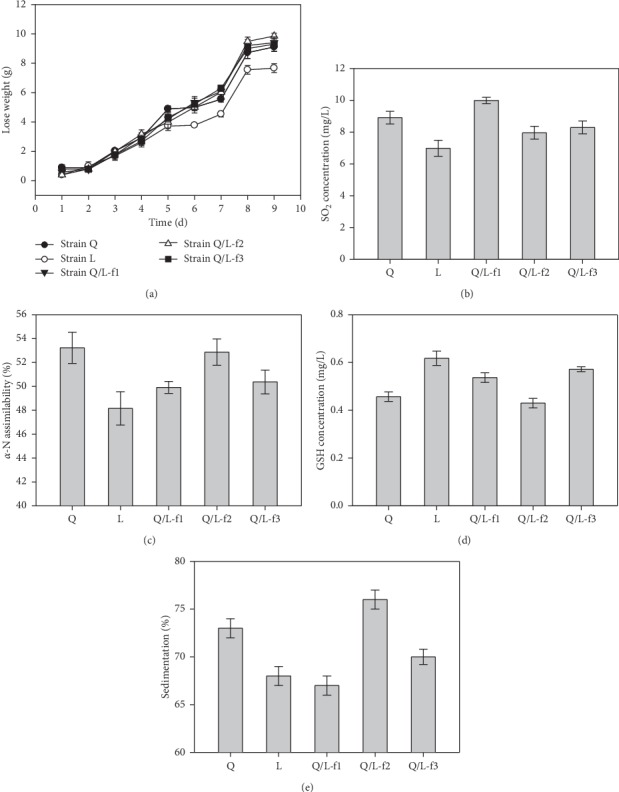
Evaluation of the fermentation properties among Q/L fusants and their parents. ((a)–(d)) Measurement of the CO_2_ weight loss (a), SO_2_ content (b), *α*-N assimilability (c), GSH content (d), and sedimentation (e) Q/L fusants and their parents. Values are presented as mean ± SD (*n* = 3).

As shown in results, strain L exhibited a higher rate of CO_2_ weight loss than strain Q, while the rate of CO_2_ weight loss is similar between strain Q and fusants (Figure 6(a)). As for SO_2_ content, the data indicated that there is few difference among strain L (9.0 mg/L), strain Q (6.986 mg/L), and three fusants (10 mg/L for Q/L-f1, 8 mg/L for Q/L-f2, 8.3 mg/L for Q/L-f3) (Figure 6(b)). The *α*-N assimilability of strain Q is 53.2%, which is higher than the counterparts of strain L (48.2%) and fusants (49.9% mg/L for Q/L-f1, 52.9% for Q/L-f2, and 50.4% for Q/L-f3) (Figure 6(c)). Meanwhile, the GSH contents of fusants are 0.54 mg/L for Q/L-f1, 0.43 mg/L for Q/L-f2 (similar to strain Q (0.46 mg/L)), and 0.57 mg/L for Q/L-f3 (similar to strain L (0.62 mg/L)) (Figure 6(d)). As for the sedimentation, three fusants were measured to be 67% (Q/L-f1), 76% (Q/L-f2), and 70% (Q/L-f3), respectively (Figure 6(e)). Therefore, the results suggested that fusant Q/L-f2 exhibits a globally similar fermentation properties while there is higher ethanol tolerance to strain Q, especially Q/L-f2 (Table 1).

Generally, the strain Q/L-f2 exhibited a 14% ethanol tolerance, which increased by 40% than the fermentative strain Q (10% ethanol tolerance). Considering the fixed ethanol content of beer (around 4% for beer), Q/L-f2 has the potential to produce more beer than its parent strain Q in the same fermentation cylinder, by increasing the ethanol content of fermentation broth. Therefore, the strain Q/L-f2 is expected to be applied into further pilot scale production of beer to decrease the manufacturing cost by around 40% than the fermentative strain Q.

## 4. Conclusion

In order to produce quality beer under high ethanol accumulation, fermenting yeast with superior ethanol tolerance while similar fermentation activity was produced. Although the fermentative strain Q and the strain L are similar in growth and genesis, L exhibited a higher ethanol tolerance (14%, v/v) than Q (10%, v/v). To produce the haploids Q-h and L-h, strains Q and L were incubated by 0.2 g/L lyticase in HS medium at 25°C for 7 days. Then, 1.5 ml 2% snailase was added into 16 h-cultured haploids under 90 min incubation at 32°C to form protoplasts Q-hp and L-hp. In order to obtain Q/L fusants, Q-hp/L-hp was inactivated by heating for 20 min/30 min at 60°C, or by UV irradiation in 30 W and 30 cm separation for 14 min. After protoplast fusion and fermentation evaluation, fusant Q/L-f2 exhibited a higher ethanol tolerance (14%, v/v) and similar fermentation properties, compared to strain Q. Thus, it was selected as candidate for further pilot scale production of beer to tremendously decrease the manufacturing cost. Our works established a series of high ethanol-tolerant strains in beer production. Moreover, this demonstration of inactivated protoplast fusion in industrial *S. cerevisiae* strain opens many doors for yeast-based biotechnological applications.

## Figures and Tables

**Table 1:** Comparison between strains Q and L and the fusant QL-f2 with respect to fermentation properties.

	Strain Q	Strain L	Fusant QL-f2
CO_2_-weight-loss rate (g·d^−1^)	1.03 ± 0.04	0.91 ± 0.08	1.18 ± 0.05
SO_2_ concentration (mg·L^−1^)	8.91 ± 0.42	6.99 ± 0.51	7.96 ± 0.44
*α*-N assimilability (%)	53.2 ± 1.31	48.2 ± 1.39	52.9 ± 1.08
GSH concentration (mg·L^−1^)	0.46 ± 0.02	0.62 ± 0.03	0.43 ± 0.02
Sedimentation (%)	73.2 ± 1.04	68.2 ± 0.98	76.6 ± 1.12
Tolerable ethanol concentration (%)	10	14	14

## Data Availability

All data used to support the findings of this study are obtained from our experiments and included within the article and/or the supplementary information file.

## Supplementary Materials

Supplementary Materials Figure S1: 26S rDNA alignment between *S. cerevisiae* strains Q and L. Query is strain Q while Sbjct is strain L. Figure S2: spore production of *S. cerevisiae* strain Q (A) and L (B) under the microscope (1000 magnification). Figure S3: released spore of *S. cerevisiae* strains Q (A) and L (B) under the microscope (1000 magnification). The spores were prestained by Carbol fuchsin and Meran carbonate.
