# Supplementary material for: Improvement of Ethanol Tolerance by Inactive Protoplast Fusion in Saccharomyces cerevisiae
Source: Biomed Res Int. 2020 Jan 20;2020:1979318. doi: 10.1155/2020/1979318 (PMC7201837; doi:10.1155/2020/1979318)

Fig. S1

```
Range 1: 1 to 613 Graphics                         Next Match   Previous Match

NW Score        Identities          Gaps             Strand
1197            610/614(99%)        2/614(0%)        Plus/Plus

Query  1      ATATCAAAAA-GCGGAGGAAAAGAAACCAACCGGGATTGCCTTAGTAACGGCGAGTGA  57
                ||||||||| |||||||||||||||||||||||||||||||||||||||||||||||
Sbjct  1    GCATATCAAAAAAGCGGAGGAAAAGAAACCAACCGGGATTGCCTTAGTAACGGCGAGTGA  60

Query  58   AGCGGCAAAAGCTCAAATTTGAAATCTGGTACCTTCGGTGCCCGAGTTGTAATTTGGAGA  117
            ||||||||||||||||||||||||||||||||||||||||||||||||||||||||||||
Sbjct  61   AGCGGCAAAAGCTCAAATTTGAAATCTGGTACCTTCGGTGCCCGAGTTGTAATTTGGAGA  120

Query  118  GGGCAACTTTGGGGCCGTTCCTTGTCTATGTTCCTTGGAACAGGACGTCATAGAGGGTGA  177
            ||||||||||||||||||||||||||||||||||||||||||||||||||||||||||||
Sbjct  121  GGGCAACTTTGGGGCCGTTCCTTGTCTATGTTCCTTGGAACAGGACGTCATAGAGGGTGA  180

Query  178  GAATCCCGTGTGGCGAGGAGTGCGGTTCTTTGTAAAGTGCCTTCGAAGAGTCGAGTTGTT  237
            ||||||||||||||||||||||||||||||||||||||||||||||||||||||||||||
Sbjct  181  GAATCCCGTGTGGCGAGGAGTGCGGTTCTTTGTAAAGTGCCTTCGAAGAGTCGAGTTGTT  240

Query  238  TGGGAATGCAGCTCTAAGTGGGTGGTAAATTCCATCTAAAGCTAAATATTGGCGAGAGAC  297
            ||||||||||||||||||||||||||||||||||||||||||||||||||||||||||||
Sbjct  241  TGGGAATGCAGCTCTAAGTGGGTGGTAAATTCCATCTAAAGCTAAATATTGGCGAGAGAC  300

Query  298  CGATAGCGAACAAGTACAGTGATGGAAAGATGAAAAGAACTTTGAAAAGAGAGTGAAAAA  357
            ||||||||||||||||||||||||||||||||||||||||||||||||||||||||||||
Sbjct  301  CGATAGCGAACAAGTACAGTGATGGAAAGATGAAAAGAACTTTGAAAAGAGAGTGAAAAA  360

Query  358  GTACGTGAAATTGTTGAAAGGGAAGGGCATTTGATCAGACATGGTGTTTTGTGCCCTCTG  417
            ||||||||||||||||||||||||||||||||||||||||||||||||||||||||||||
Sbjct  361  GTACGTGAAATTGTTGAAAGGGAAGGGCATTTGATCAGACATGGTGTTTTGTGCCCTCTG  420

Query  418  CTCCTTGTGGGTAGGGGAATCTCGCATTTCACTGGGCCAGCATCAGTTTTGGTGGCAGGA  477
            ||||||||||||||||||||||||||||||||||||||||||||||||||||||||||||
Sbjct  421  CTCCTTGTGGGTAGGGGAATCTCGCATTTCACTGGGCCAGCATCAGTTTTGGTGGCAGGA  480

Query  478  TAAATCCATAGGAATGTAGCTTGCCTCGGTAAGTATTATAGCCTGTGGGAATACTGCCAG  537
            ||||||||||||||||||||||||||||||||||||||||||||||||||||||||||||
Sbjct  481  TAAATCCATAGGAATGTAGCTTGCCTCGGTAAGTATTATAGCCTGTGGGAATACTGCCAG  540

Query  538  CTGGGACTGAGGACTGCGACGTAAGTCAAGGATGCTGGCATAATGGTTATATGCCGCCCG  597
            ||||||||||||||||||||||||||||||||||||||||||||||||||||||||||||
Sbjct  541  CTGGGACTGAGGACTGCGACGTAAGTCAAGGATGCTGGCATAATGGTTATATGCCGCCCG  600

Query  598  TCTTGAAACACGGG  611
            |||||||||||||
Sbjct  601  TCTTGAAACACGG   613
```

Fig. S2

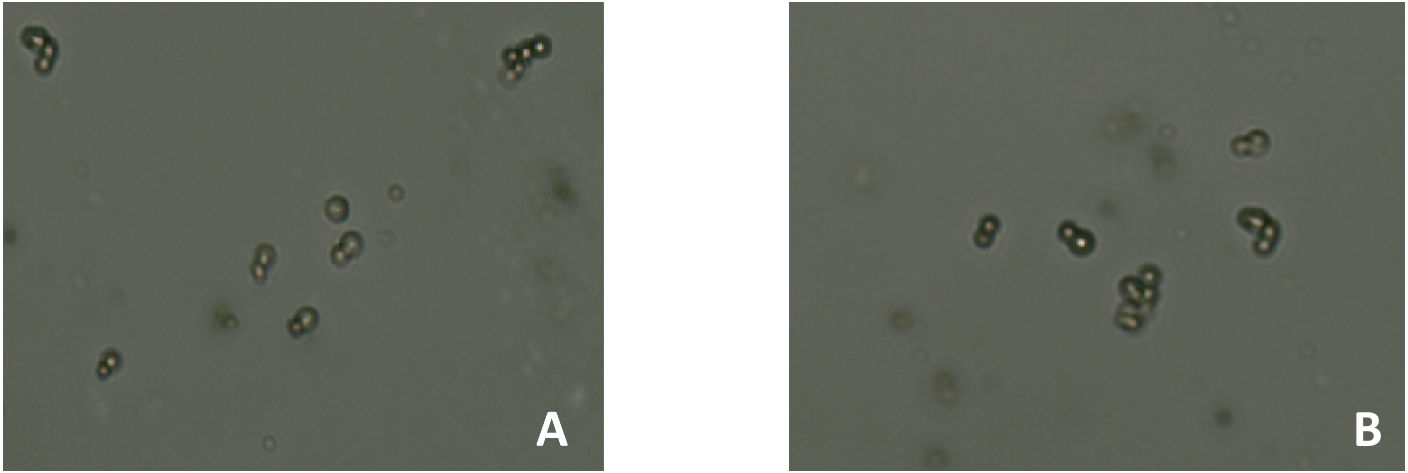

Fig. S3

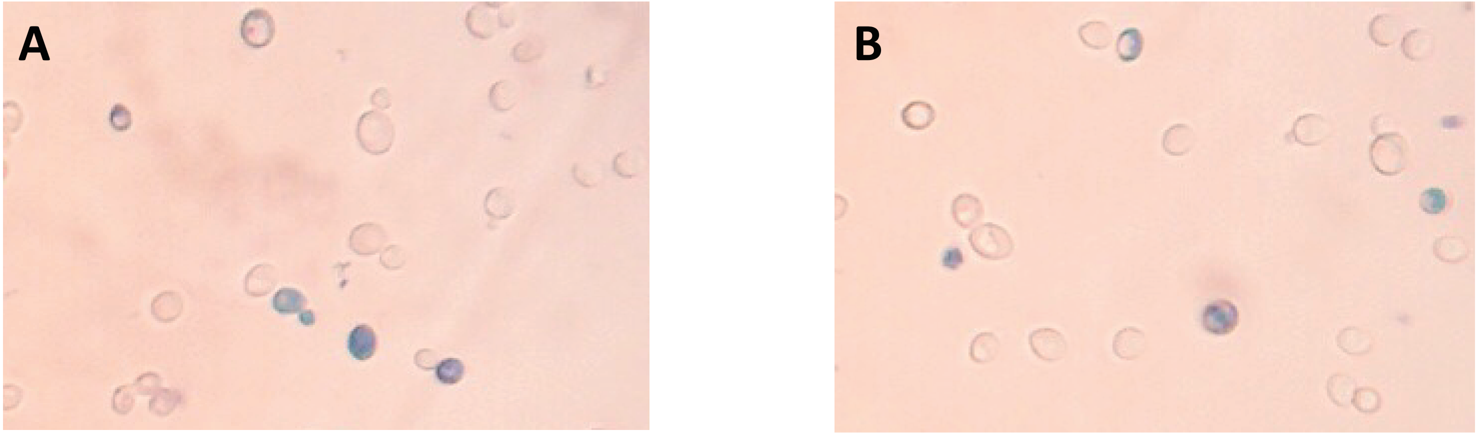

Supplement: Supplementary Materials — Figure S1: 26S rDNA alignment between S. cerevisiae strains Q and L. Query is strain Q while Sbjct is strain L. Figure S2: spore production of S. cerevisiae strain Q (A) and L (B) under the microscope (1000 magnification). Figure S3: released spore of S. cerevisiae strains Q (A) and L (B) under the microscope (1000 magnification). The spores were prestained by Carbol fuchsin and Meran carbonate. [file 1979318.f1.pdf]
